# 3D Printing of Naturally Derived Adhesive Hemostatic Sponge

**DOI:** 10.34133/research.0446

**Published:** 2024-08-09

**Authors:** Minyu Zhou, Tao Yuan, Luoran Shang

**Affiliations:** ^1^ The First Affiliated Hospital of Wenzhou Medical University, Wenzhou 325035, China.; ^2^ Department of Spine Surgery, Second Xiangya Hospital of Central South University, Changsha 410011, China.; ^3^ Shanghai Xuhui Central Hospital, Zhongshan-Xuhui Hospital, and the Shanghai Key Laboratory of Medical Epigenetics, the International Co-laboratory of Medical Epigenetics and Metabolism (Ministry of Science and Technology), Institutes of Biomedical Sciences, Fudan University, Shanghai, China.

## Abstract

Hydrogel hemostatic sponges have been recognized for its effectiveness in wound treatment due to its excellent biocompatibility, degradability, as well as multi-facet functionalities. Current research focuses on optimizing the composition and structure of the sponge to enhance its therapeutic effectiveness. Here, we propose an adhesive hydrogel made from purely natural substances extracted from okra and Panax notoginseng. We utilize 3-dimensional (3D) printing technology to fabricate the hemostatic hydrogel scaffold, incorporating gelatin into the hydrogel and refining the mixing ratio. The interaction between gelatin and okra polyphenols contributes to successful injectability as well as stability of the printed scaffold. The okra in the scaffold exhibits favorable adhesion and hemostatic effects, and the total saponins of Panax notoginseng facilitate angiogenesis. Through in vitro experiments, we have substantiated the scaffold's excellent stability, adhesion, biocompatibility, and angiogenesis-promoting ability. Furthermore, in vivo experiments have demonstrated its dual functionality in rapid hemostasis and wound repair. These features suggest that the 3D-printed, natural substance-derived hydrogel scaffolds have valuable potential in wound healing and related applications.

## Introduction

Millions of individuals worldwide experience skin trauma [1,2], and over 30% of traumatic fatalities are linked to uncontrolled bleeding [3–5]. While bleeding wounds represent a commonplace physiological occurrence, their treatment merits special attention [6–12]. Currently, a lot of methods have been developed for achieving rapid hemostasis, among which the use of hydrogel materials has demonstrated several advantages including swift hemostasis [13–15], ease of use [16], and versatile morphology [17–20], etc. [21–24]. Hemostatic sponges can be prepared by selecting different materials based on specific requirements such as controllable water absorption, anti-infection, and tissue adhesion [25–28]. Although promising results have been achieved, the complex preparation steps and undefined biodegradability of synthetic polymer materials pose challenges to their practical use, and there is still potential for enhancement regarding bioactive properties to meet the requirement for tissue regeneration [29–34]. Therefore, novel hemostatic sponges with better bioactive properties as well as a simple preparation process are highly expected.

**Fig. 1.**
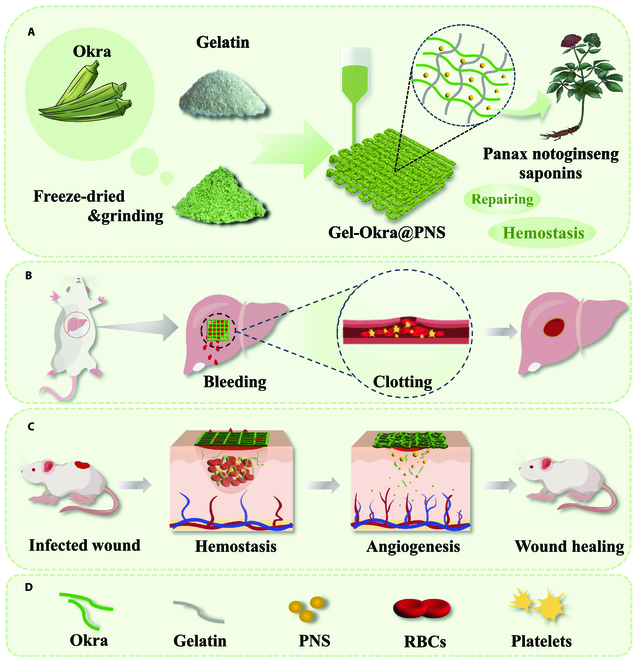
Schematic illustration of the preparation of PNS-loaded okra-gelatin gel scaffold (Gel-Okra@PNS) by 3D printing for liver hemostasis and skin wound repair. (A) Design of the main contents of the Gel-Okra@PNS hydrogel scaffold and the 3D printing process. (B) Hemostatic effect of Gel-Okra@PNS scaffold on a rat liver hemorrhage wound model. (C) Effect of Gel-Okra@PNS scaffold on promoting neovascularization and collagen deposition on bleeding wounds. (D) Legend for the above element.

In this paper, we propose 3-dimensional (3D)-printed adhesive hemostatic sponge derived from natural substances, as shown in Fig. 1. Natural products exhibit a wide range of functions. Specifically, okra mucilage contains abundant polysaccharides, including galactose, rhamnose, and galacturonic acid [35]. The high polysaccharide content makes it sticky and renders okra gel hemostatic properties, i.e., it can form a protective layer over wounds. Additionally, okra is rich in nutrients such as trace elements and vitamins, some of which are essential for blood clotting [36–40]. Besides, Panax notoginseng saponins (PNS), a naturally occurring compound derived from the Panax notoginseng plant, play a pivotal role in promoting angiogenesis and wound healing [41–46]. Alternatively, 3D printing technology enables rapid fabrication of hydrogel scaffolds with time and labor efficiency, as well as meticulous regulation of the scaffold's shape and structure [47–49]. The vision is that by leveraging 3D printing technology, natural bioactive hydrogel sponges can be constructed for hemostasis.

Here, we prepared 3D-printed okra gel-based scaffold with the incorporation of PNS as an adhesive hemostatic sponge. By leveraging the favorable rheological properties of gelatin, as well as the binding of gelatin to functional groups in okra [50–55], such as okra polyphenols, the hydrogel can be easily extruded while exhibiting high shape fidelity after printing [56]. Additionally, PNS was loaded into the scaffold, which imparted the scaffold with angiogenesis-promoting and wound healing-promoting capabilities. Based on these properties, we demonstrated the fast hemostatic ability of the okra-based hydrogel scaffold in bleeding wounds. Additionally, the scaffold can conform and adhere well to irregularly shaped wounds. Furthermore, the natural substance-derived scaffold exhibited good biodegradability, allowing for its effective removal after use with minimized risk of secondary bleeding. These results demonstrated that the present okra hydrogel scaffold holds great promise for achieving rapid hemostasis and promoting the healing of bleeding wounds [21,57]. We believe that the method of 3D printing of natural bioactive hydrogels has great potential applications in the biomedical field.

## Results

**Fig. 2.**
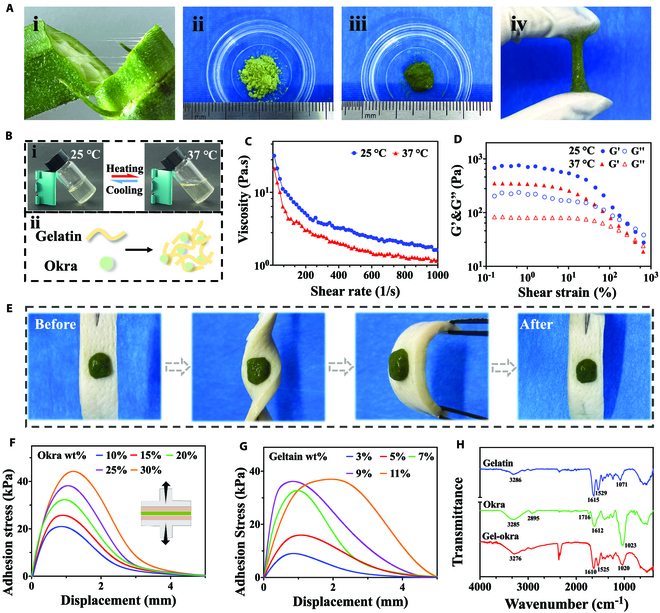
The rheological and mechanical properties of Gel-Okra gel. (A) (i) A piece of okra with the mucus. (ii) Freeze-dried okra powder. (iii) Okra gel (formed by 20 mg of okra powder dissolved in 100 μl of water). (iv) Strong adhesion of the okra gel. (B) (i) Gelatin remained in gelling state at 25 °C and became liquid at 37 °C. (ii) Diagram of crosslinking between gelatin and okra. (C) Viscosity measurements of Gel-Okra as a function of the shear rate at different temperatures. (D) Amplitude sweep tests on Gel-Okra gels with different concentrations of gelatin as the applied strain increased from 0.1% to 1,000% at different temperatures. (E) Photographs showing that the Gel-Okra gel adhered firmly to the pigskin after being twisted and bent. (F) Measuring the adhesion of Gel-Okra to pigskin by a universal testing machine. The gelatin powder concentration was 7%. (G) Measuring the adhesion of Gel-Okra to pigskin by a universal testing machine. The okra powder concentration was 20%. (H) Fourier transform infrared spectra of freeze-dried powder of okra, gelatin, and Gel-Okra gel.

Okra possesses abundant mucilage. After freeze-drying and grinding it into powder, we rehydrated it with water to achieve a highly viscous and adhesive okra gel (Fig. 2A). We found that the okra gel filaments extruded from a needle showed a tendency to merge (Fig. S1). Therefore, to optimize the printability, gelatin was employed as a supramolecular gelling agent. Gelatin displayed gel-to-sol transition when the temperature increased, as shown in Fig. 2B. Additionally, okra’s polyphenols can serve as cross-linking agents for gelatin. After combining gelatin and okra, we observed in infrared spectra that the amide bond peak near 3,300^−1^ shifted and widened, suggesting that hydrogen bonding may be at play (Fig. 2H). Therefore, we mixed okra powder with gelatin to prepare Gel-Okra gel (Fig. 2B). Rheological tests were performed. As shown in Fig. 2C, the viscosity of the Gel-Okra gel decreased gradually with increasing shear rate. Such shear-thinning property can be favorable for printing. In addition, at higher temperatures, the gel tends to have a lower viscosity and flow more easily through the printing channel. Subsequently, amplitude sweep tests were conducted on Gel-Okra gels (Fig. 2D). We found that as the strain increases, there is a crossover between *G*′ and *G*″, indicating the transition into a viscoelastic liquid state that facilitates successful injectability.

Since okra’s polyphenols can function as bioadhesives, we next evaluated the tissue adhesion ability of the Gel-Okra gel. A gel block was placed on the surface of the porcine skin. As shown in Fig. 2E, despite being subjected to twisting and bending, the gel retained its integrity and firmly adhered to the tissue. These findings validated the efficacy of the Gel-Okra gel as tissue adhesives in dynamic environments. Tensile tests were conducted to quantitatively evaluate the strength of the gel in adhering to pigskin. The results demonstrated that the strength increased with increasing concentrations of okra or gelatin (Fig. 2F and G).

**Fig. 3.**
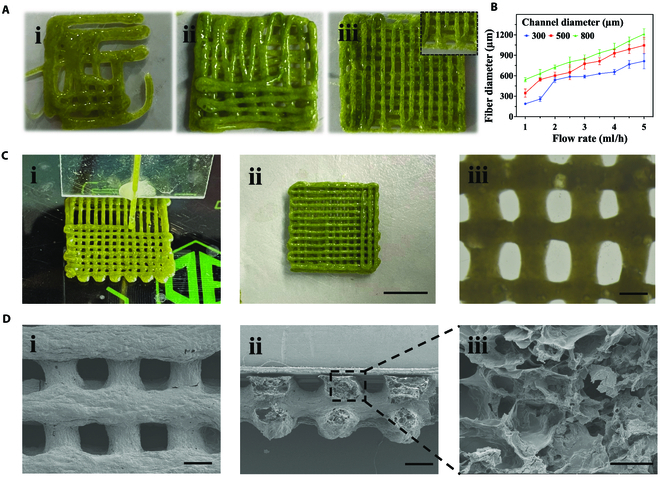
Characterization of Gel-Okra scaffolds. (A) Photographs of 3D-printed scaffolds composed of different concentrations of okra and gelatin: (i) 5% gel + 15% okra; (ii) 15% gel + 15% okra; (iii) 10% gel + 20% okra. (B) Effect of the capillary channel diameter and flow rate on the diameter of the constituent fiber of the scaffold. (C) (i) Process of 3D printing of the Gel-Okra hydrogel scaffold. (ii) Photograph of a printed scaffold. (iii) Optical microscopic image of a printed Gel-Okra scaffold. (D) Scanning electron microscopy (SEM) images of freeze-dried Gel-Okra scaffolds at (i) top-down and (ii and iii) cross-sectional views. Scale bars, 1 cm (Cii), 500 μm (Ciii, Di, and Dii), and 50 μm (Diii).

Given the proper rheological and adhesion properties of the Gel-Okra gel, we next employed the 3D printing technique to fabricate Gel-Okra@PNS scaffold. Specifically, okra powder was first dispersed in a gelatin solution, where the polyphenols in okra interacted with gelatin to form a gel. Using a programmable 3D printing platform, hydrogel fibers were extruded from a capillary device with a tapered tip (Fig. S2). The photographs of scaffolds printed with different concentrations of okra and gelatin compositions are presented in Fig. 3A. Inadequate okra concentration results in a diluted solution unfavorable for shape formation, whereas excessive concentration elevates the resistance during extrusion. Additionally, insufficient gelatin concentration leads to susceptibility to fusion of the printed filament, whereas excessive gelatin concentration amplifies resistance and impedes fiber extrusion. With this, the optimized composition of the printing ink was selected as 7% gelatin and 20% okra, through which a well-formed scaffold can be printed smoothly. An optimal match between fluid flow rate and printhead motion speed was employed such that the microfibers were gradually stacked on a dry Petri dish to form a 3D scaffold (Fig. 3C). In addition, the diameter of the constituent fiber of the scaffold can be adjusted by tuning the capillary tube diameter and injection flow rate, as shown in Fig. 3B. The resulting scaffolds were then subjected to an overnight freezing process at −80 °C and then freeze-dried. The scanning electron microscopy (SEM) images demonstrated the porous structure of the scaffold (Fig. 3D). The printed scaffold also has good adhesion capabilities (Fig. S3).

**Fig. 4.**
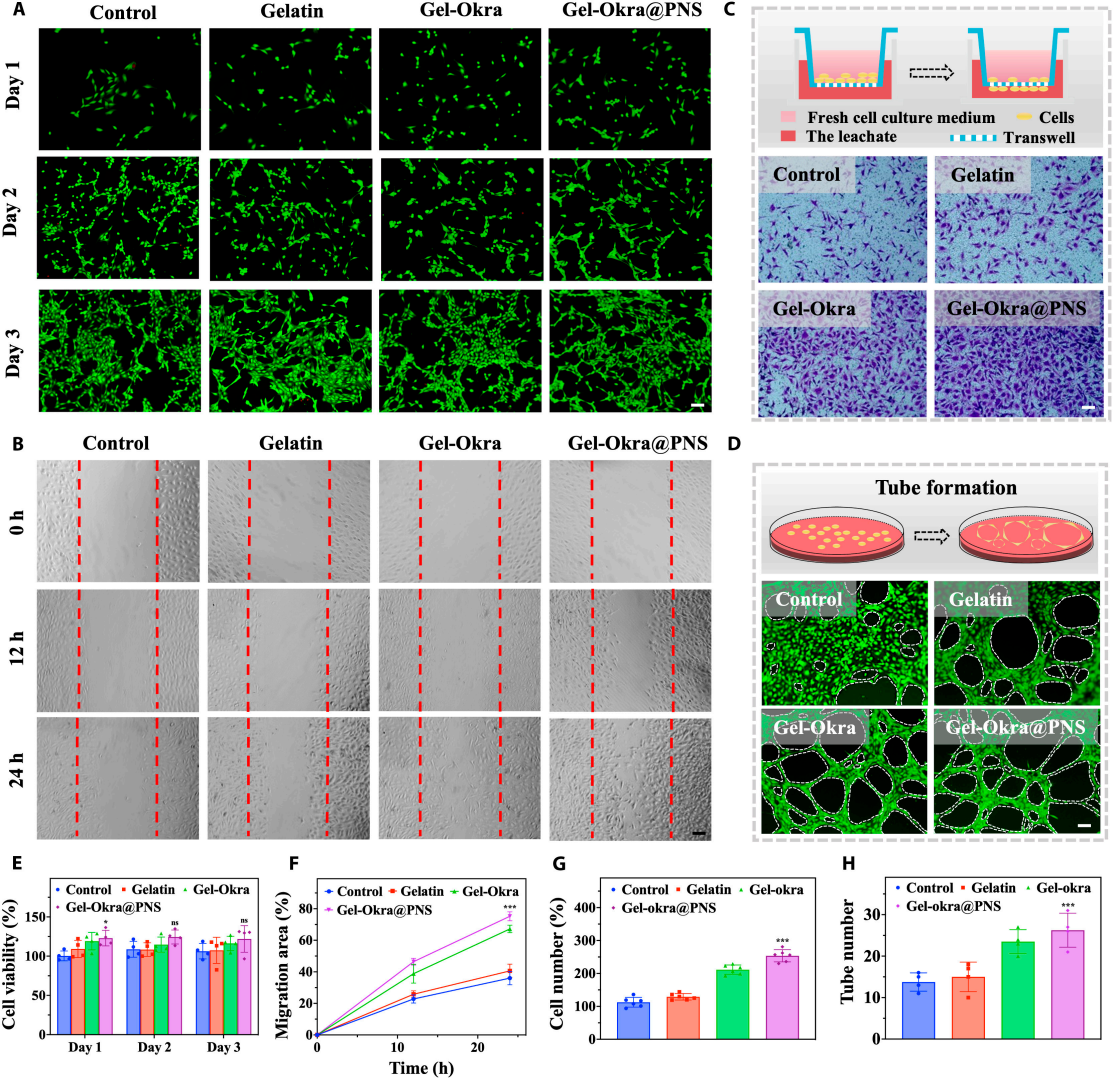
In vitro cell experiments of scaffolds. (A and E) Representative visuals and quantitative assessment of live/dead staining for 3T3 cultured with gelatin, Gel-Okra, and Gel-Okra@PNS scaffold extracts. Fluorescence is utilized to distinguish cells between live (green) and dead (red). (B and F) Illustrative visuals and quantitative analysis of in vitro scratch experiments on HUVECs. Dashed lines delineate the initial edges of the scratch. (C and G) Illustrative images alongside quantitative analysis of the assay of HUVEC transwell migration. (D and H) Representative visuals and quantification of HUVEC tube formation. The dashed circles outline the developed tubes. All scale bars indicate 100 μm. *P < 0.01, **P < 0.01, ***P < 0.001, and ns, not significant; compared with the Control group.

To assess the biocompatibility of the printed scaffolds, we observed significant proliferative behavior of 3T3 cells that were incubated with gelatin, Gel-Okra, and Gel-Okra@PNS scaffold extracts and cultured for 3 d. The results confirmed the biocompatibility of the scaffolds (Fig. 4A and E). We then assessed the cytotoxicity of hydrogels with varying concentrations of Gel-Okra@PNS and found high cell viability over the 3-d culture period (Fig. S4), demonstrating the nontoxic nature of Gel-Okra@PNS as the component of the scaffold. To investigate whether Gel-Okra@PNS scaffolds can enhance cell migration for human umbilical vein endothelial cells (HUVECs), we conducted a standard scratch experiment. The results indicated an accelerated wound closure rate in the Gel-Okra@PNS group compared to the other groups (Fig. 4B and F). Additionally, the transwell migration assay of HUVECs confirmed that the Gel-Okra@PNS scaffold increased cell mobility (Fig. 4C and G). Moreover, the release behavior of PNS from the Gel-Okra@PNS scaffolds was investigated (Fig. S5). PNS can be gradually released from the scaffolds. We reasoned that the sustained release of PNS from the scaffolds can promote angiogenesis. To validate this, we conducted Matrigel tube formation experiments, which revealed a higher number of vessel-like tubes in the Gel-Okra@PNS group compared to the other groups after 6 h (Fig. 4D and H).

**Fig. 5.**
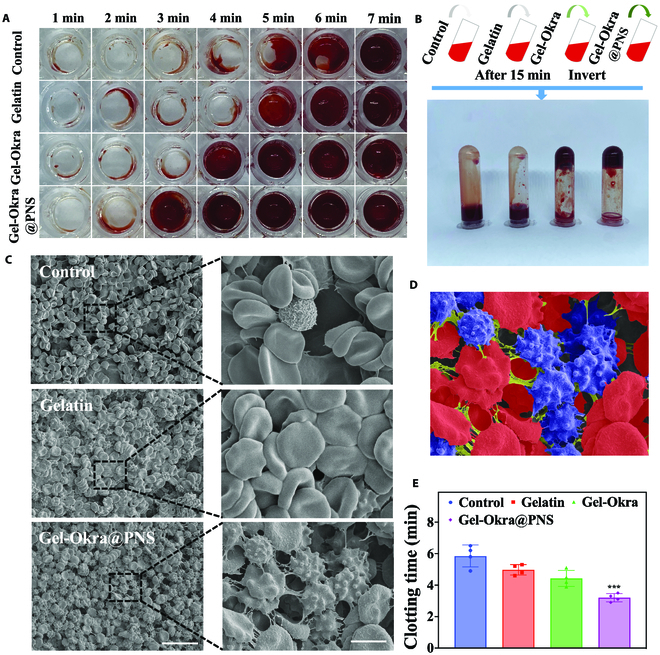
The in vitro blood coagulation assays and SEM of activated platelets. (A) Images depicting clot formation over time. (B) Photos of coagulation conditions after mixing normal whole blood, blood sample mixed with gelatin, Gel-Okra scaffold powder, and Gel-Okra scaffold powder for 15 min. (C) SEM images of gelatin, Gel-Okra scaffold, and Gel-Okra@PNS scaffold mixed with whole blood at different magnifications. (D) SEM image illustrating red blood cells (red), platelets (blue), and Gel-Okra scaffold (green). (E) Quantitative data of the clotting time in each group. Scale bars, 25 μm (C, left) and 5 μm (C, right). *P < 0.01, **P < 0.01, ***P < 0.001, and ns, not significant; compared with the Control group.

To investigate the hemostasis role of the Gel-Okra@PNS scaffold, we performed an in vitro coagulation test. Under normal conditions, blood typically starts to clot in 5 to 6 min (Fig. 5A to E). The gelatin group exhibited a slight acceleration in the hemostatic process, whereas the Gel-Okra and Gel-Okra@PNS groups formed substantial blood clots in 3 and 4 min, respectively. Subsequently, we added 5 mg of gelatin, Gel-Okra, and Gel-Okra@PNS powder to tubes containing 1 ml of human blood specimens with the anticoagulant. The tubes were gently inverted multiple times and then allowed to stand at room temperature for 15 min (Fig. 5B). In the Gel-Okra and Gel-Okra@PNS groups, a significant amount of blood formed a clot at the bottom of the tube, indicating an intense hemagglutination response. Within the control group and the gelatin group, blood flowed freely. Significant differences were observed among groups based on coagulation indices (Table S1). The results indicated that okra has a coagulation-promoting effect in vitro. According to previous studies, the hemostatic mechanism of okra primarily involves platelet activation [36]. To illustrate this, SEM images were taken for the hydrogel scaffolds mixed with whole blood. Relative to the control group, where the platelets were resting, the platelets in the Gel-Okra@PNS group displayed a more irregular spiny shape, suggesting the activated state (Fig. 5C and D and Fig. S6) [58].

**Fig. 6.**
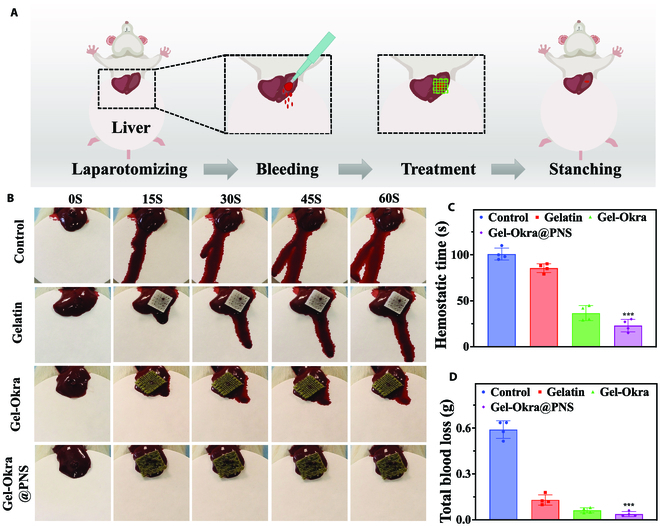
In vivo hemostatic ability of Gel-Okra@PNS scaffold. (A) Schematic illustration of the hemostasis of rat liver wounds using Gel-Okra@PNS. (B) Photographs showing liver hemostasis with different treatments in each group. (C) Hemostasis time in different groups. (D) Total blood loss (*n* = 4). *P < 0.01, **P < 0.01, ***P < 0.001, and ns, not significant; compared with the Control group.

Based on this, we investigated the in vivo hemostatic capability of Gel-Okra@PNS using a standard rat liver hemorrhage wound model, as depicted in Fig. 6A and B. In the group of Gel-Okra@PNS, bleeding diminished rapidly and came to a complete stop, with minimal blood stains on the filter paper. However, the control group experienced uncontrolled bleeding. Quantitative analysis revealed that the hemostasis time of Gel-Okra@PNS and Gel-Okra was notably shorter compared to that of the remaining 2 groups (Fig. 6C). Additionally, the total blood loss in the Gel-Okra@PNS and Gel-Okra groups was markedly lower in the remaining 2 groups (Fig. 6D). Notably, the Gel-Okra@PNS group exhibited the shortest hemostasis time and least blood loss among all groups, confirming the synergistic coagulation effect of Okra and PNS.

**Fig. 7.**
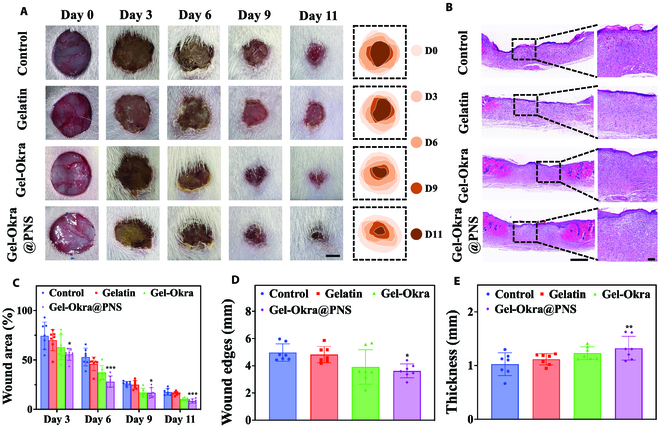
In vivo wound healing capacity of Gel-Okra@PNS scaffolds. (A) Photographs of skin wounds after 11 d of different treatments and relative changes in wound area. (B) Hematoxylin and eosin staining images on day 11. (C) Quantitative analysis of the wound area on day 11 (*n* = 7). (D) Measurement of wound width at day 11 (*n* = 7). (E) Quantitative measurement of dermis thickness of wounds on the 11th day. Scale bars, 5 mm (A), 1 mm (B, left), and 100 μm (B, right). *P < 0.01, **P < 0.01, ***P < 0.001, and ns, not significant; compared with the Control group.

We conducted further investigations to assess the in vivo wound healing capacity of Gel-Okra@PNS scaffolds using a rat skin wound model (Fig. S7). As shown in Fig. 7A and C, wound area gradually decreased in all groups over time, but the Gel-Okra@PNS group exhibited the most effective wound closure among all groups, in sharp contrast to the phosphate-buffered saline (PBS) and gelatin groups. Tissue regeneration was further assessed by histological analysis, as shown in hematoxylin and eosin (H&E) staining results, which revealed that the Gel-Okra@PNS group exhibited thicker regeneration tissue and smaller wound edges compared to other groups (Fig. 7B, D, and E).

**Fig. 8.**
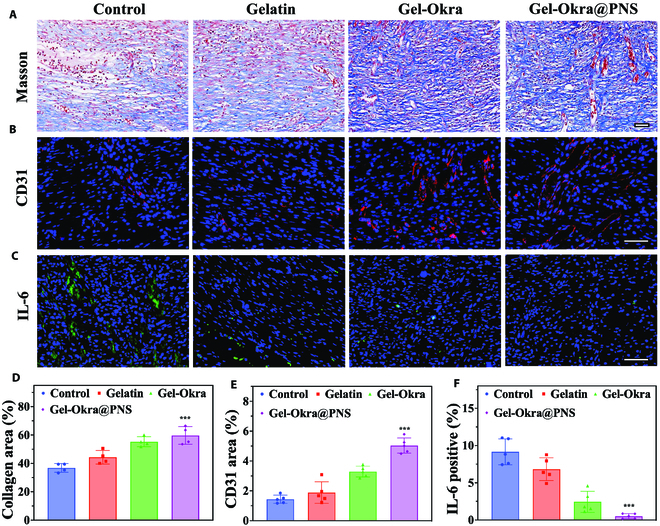
Wound histopathological analyses on day 11. (A) Images depicting Masson staining of wounds in various groups. (B) Immunofluorescent staining (red fluorescence) for CD31. (C) Immunohistochemical staining for IL-6 (red fluorescence). (D) Measurement of collagen deposition area. (E) Quantification of CD31. (F) Expression of IL-6 in different groups. All scale bars are 50 μm. *P < 0.01, **P < 0.01, ***P < 0.001, and ns, not significant; compared with the Control group.

Masson's trichrome staining was employed to visualize deposition of collagen at the wound site (Fig. 8A and D). The Gel-Okra@PNS and Gel-Okra groups revealed higher collagen deposition compared to the others. Considering the known role of PNS saponins in promoting angiogenesis, we performed immunofluorescence staining for CD31 to assess the impact of the scaffold on angiogenesis. As depicted in Fig. 8B and E, the Gel-Okra@PNS group demonstrated the highest expression level of CD31. Moreover, the inflammation marker interleukin-6 (IL-6) in each group was examined. As shown in Fig. 8C and F, the expression in the Gel-Okra@PNS group was the lowest. In summary, these findings collectively indicated that the Gel-Okra@PNS hydrogel scaffold has the prospective to greatly improve wound healing by promoting neovascularization, facilitating collagen deposition, and reducing inflammation.

## Discussion

In this research, we proposed a natural material hydrogel scaffold (Gel-Okra@PNS) through 3D printing for rapid hemostasis and promotion of wound healing. The scaffold was composed of okra, gelatin, and total saponins of Panax notoginseng. Okra has an adhesive effect and contains various bioactive components, which promote hemostasis and wound healing. The addition of gelatin facilitates a smoother printing process. The total saponins of Panax notoginseng are beneficial to angiogenesis. With these properties, the Gel-Okra@PNS scaffold showed significant tissue adhesion, procoagulation, cell migration, and tube formation, as confirmed by in vitro experiments. In in vivo experiments, we also showed procoagulant and rapid hemostasis functions and enhanced neovascularization and collagen formation during wound healing. In addition, customized microfluidic 3D bioprinting platforms can be further adapted to create patient-specific wound dressings with the required geometry, composition, and functionality for various wound types [59–61]. These results validated the great promise of the scaffold for applications in rapid hemostasis and tissue repair.

However, the combination of natural materials and 3D printing technology needs to pass strict preclinical and clinical testing and comply with relevant medical device regulations and standards. Overall, greater efforts need to be made to apply this hemostatic scaffold to clinical settings.

## Materials and Methods

### Materials

Gelatin was obtained from Shanghai Aladdin Co. Ltd., China. PNS was procured from Beijing Solarbio Technology Co. Ltd. Okra was purchased from a local vegetable market. HUVECs and endothelial cell medium (ECM) were acquired from ScienCell. Cell Counting Kit-8 (CCK-8) was acquired from Beyotime Biotechnology Co. Ltd. Sprague Dawley rats were provided by Beijing Vital River Laboratory Animal Technology Co. Ltd. Ultrapure water (18.2 MΩ cm^−1^, Millipore) was used throughout the experiments. All experimental protocols mentioned in this article have received approval from the institution where they are conducted (wiucas23033001).

### Characterization

The optical photographs of the scaffolds were captured by Olympus SZX-16. SEM (SU8010, Hitachi) was employed to observe the microstructure of the scaffold. The adhesive strength of the gel was evaluated using an electronic universal testing machine (5944, Instron). A rheometer was utilized to assess the rheological properties of each gel sample. Fluorescence images were screened by Axio Vert.A1.

### Preparation of Gel-Okra@PNS scaffold and its extract

A fluid injection chip was assembled with a glass slide and a cylindrical glass capillary. The capillary was tapered with an orifice diameter of 580 to 600 μm and was fixed on a glass slide. The printing feedstock was prepared by mixing okra (20% w/v), gelatin (7% w/v), and PNS (1 mg/ml) with deionized water. The gel was injected via the chip using a programmed syringe pump at an appropriate flow rate (4 ml/h). The 3D printer was set to a moving speed of 3 mm/s. The syringe outlet is heated to facilitate the smooth passage of the gel through the tube. As the gel moves, its temperature gradually decreases. Eventually, the filaments emerging from the chip outlet can be collected and stacked in a dry Petri dish to form a scaffold. The as-prepared sample was frozen at −80 °C. Finally, Gel-Okra@PNS scaffolds were obtained through lyophilization.

### In vitro drug release assays

Gel-Okra@PNS scaffolds (20 mm × 20 mm × 2 mm) were immersed in 2 ml pf PBS at 37 °C. PNS concentration was determined by measuring absorbance at 205 nm using an ultraviolet (UV)-visible spectrophotometer. The drug release percentage was calculated using the following formula: drug release (%) = (cumulative released drug amount/total drug amount) × 100%.

### The adhesion strength between the skin tissue and Gel-Okra gel

A piece of porcine tissue was placed on a horizontal platform. The surface of the gel was wetted with a PBS solution and then brought into contact with the skin tissue for 1 min under a pre-pressure of 5 N. The pressure sensor located on the upper side was pulled upward at a velocity of 20 mm/min. The stretching process was manually terminated when the gel separated from the skin tissue. The maximum pulling force can be obtained from the force–displacement curve.

### Rheology test

Rheological tests were conducted using a DHR-2 rotational rheometer. Viscosity test was measured through the shear rate sweep from 0.1 to 1,000 s^−1^. At an unchanging angular frequency of 10 rad s^−1^, the rheological amplitude sweep tests were experimented with increasing applied strain increasing from 0.1 to 1,000%.

### Scaffolds' cytotoxicity evaluation

Cytotoxicity of okra gel powder was assessed using CCK-8. Fibroblasts (3T3) were added in 96-well plates at 1 × 10^3^ cells per well. Then, it was in a cell culture incubator for 24 h subsequently. Cells were cultured in each group of hydrogel leachates for 1, 2, and 3 d. The sample solution is discarded and replaced with a mixture of CCK-8 reagent and fresh culture medium in a 1:9 ratio. The mixture was subsequently incubated in the incubator for an additional 2 h. The microplate reader was set to 450 nm, and absorbance was measured. Each sample underwent testing in 4 independent cultures, and the cytotoxicity assessment was repeated 3 times.

### Cell scratch healing assay

HUVECs were cultured in 24-well plates of 1 × 10^5^ cells per well. Then, each well is supplemented with either fresh cell culture medium or the leachate of each group of materials, followed by overnight incubation. A single scratch was utilizing a 200-μl pipette tip after a 12-h incubation. The wells were washed twice with PBS to eliminate unattached cells. Then, leaching solution specific to each group of scaffolds was replaced, and each group was incubated individually. Images were captured at regular intervals to determine the relative migration area, calculated as (1 − *W*/*W*_0_) × 100%, where *W* symbolizes the wound area at specific time points and *W*_0_ symbolizes the wound area after scraping.

### Transwell migration assay

HUVECs were cultured in transwell inserts with filters of 8-μm pore size. Cells (1 × 10^4^) were dropped into the upper chamber, while 600 μl of scaffold leachate was loaded in the lower layer, and the cells were continued to be cultured for 1 d. After the culture medium was discarded, the cells were fixed with an appropriate amount of paraformaldehyde. Cells on the upper surface were carefully wiped away. The cells that moved to the bottom of the filter were stained with 0.1% crystal violet, and pictures were taken (Nikon, Japan).

### Tube formation assay

The 24-well plate was precoated with Matrigel matrix (BD, USA). Then, each well was seeded with 5 × 10^4^ HUVECs per well with 250 μl of culture medium. The cells were exposed to various groups of scaffold leachate for a duration of 6 h and subsequently observed under a microscope. After staining with calcein-AM, the tube formation of HUVECs was visualized using a fluorescence microscope (Zeiss).

### Coagulation time test

Four groups of scaffold materials were pulverized into powder and weighed. Dispense into each well of a 96-well plate at 5 mg per well. Citrated blood was transferred to an Eppendorf tube, followed by the addition of calcium chloride (0.1 M) at 1:9 ratio (calcium chloride:citrated blood). The mixture was agitated using a vortex shaker for 10 s. Subsequently, 50 μl was dispensed into each well of the 96-well plate, and then the plate was allowed to rest during the subsequent clotting process. The coagulation process was interrupted by rinsing each well with saline every minute for a total duration of 7 min. The wells were rinsed repeatedly until the solution became clear. The final clotting time was recorded in wells where uniform clot formation occurred after completing the test.

### Investigation of platelet activation

The mechanism of scaffolds to enhance blood coagulation via platelet activation was assessed by comparing the morphological alterations of platelets when they were combined with gelatin, Gel-Okra scaffold, or Gel-Okra@PNS scaffold in vitro. Each set of scaffolds (40 mg) was incubated with 1 ml of blood sample for 1 min. The samples were fixed with 4% glutaraldehyde for 12 h. After, the samples were rinsed with PBS and subsequently dehydrated in different concentrations of ethanol for 5 min each time. To observe the morphological alterations of activated platelets, the samples used a sputter coater to coat with gold and then examined using SEM.

### In vivo hemostasis in rat liver

Female Sprague Dawley rats were used, and the liver lobes were selected. A wound with a depth of 2 mm was created on the liver of the Sprague Dawley rat using a circular blade (Φ8 mm). The hydrogel scaffold was applied to the incision, and observations were continued for 2 min.

### In vivo wound healing in rats

The 7- to 8-week-old female Sprague Dawley rats, weighing 180 to 200 g. A 15-mm-diameter skin wound was created on the back of each shaved rat using surgical scissors. The hydrogel scaffold was promptly applied to cover the wound. The daily skin wound area was documented using a camera and then computed as the wound area = *W*_t_ /*W*_0_ × 100%, where *W*_t_ represents the wound area on day *t* (*t* = 0, 3, 6, 9, and 11) and *W*_0_ represents the wound area immediately after injury. All rats were sacrificed, and tissue samples were collected on day 11. The histological analysis involved standard H&E and Masson’s staining. Immunohistochemical analysis of CD31 and IL-6 was also performed to evaluate vascularity and inflammation within the regenerated skin tissue. Tissue examinations were carried out with a fluorescence microscope.

### Statistical analysis

The above data were expressed as mean ± SD. Statistical significance between single groups was analyzed by ordinary one-way analysis of variance (ANOVA).

## Data Availability

All data are contained in the manuscript text and Supplementary Materials.
