# Supplementary material for: 3D Printing of Naturally Derived Adhesive Hemostatic Sponge
Source: Research (Wash D C). 2024 Aug 9;7:0446. doi: 10.34133/research.0446 (PMC11309851; doi:10.34133/research.0446)
Supplement: Supplementary 1 — Figs. S1 to S7 Table S1 [file research.0446.f1.docx]

Title

3D Printing of Naturally Derived Adhesive Hemostatic Sponge

**Authors**

Minyu Zhou^1^, Tao Yuan^2^, Luoran Shang^1, 3^*

**Affiliations**

^1^ The First Affiliated Hospital of Wenzhou Medical University, Wenzhou 325035, China

^2^ Department of Spine Surgery, Second Xiangya Hospital of Central South University, Changsha 410011, China

^3^ Shanghai Xuhui Central Hospital, Zhongshan-Xuhui Hospital, and the Shanghai Key Laboratory of Medical Epigenetics, the International Co-laboratory of Medical Epigenetics and Metabolism (Ministry of Science and Technology), Institutes of Biomedical Sciences, Fudan University, Shanghai, China.

Correspondence should be addressed to Luoran Shang; [luoranshang@fudan.edu.cn](mailto:luoranshang@fudan.edu.cn)


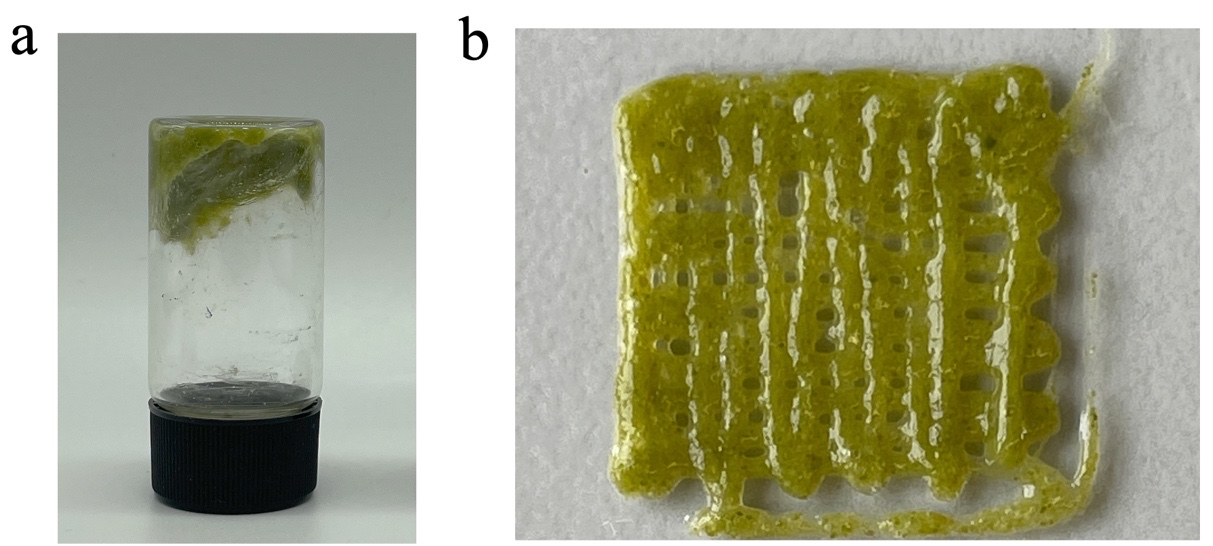


***Figure S1.*** *(a) 20% Okra gel; (b) a scaffold formed with 20% okra gel, in which the printed filaments merged.*

**
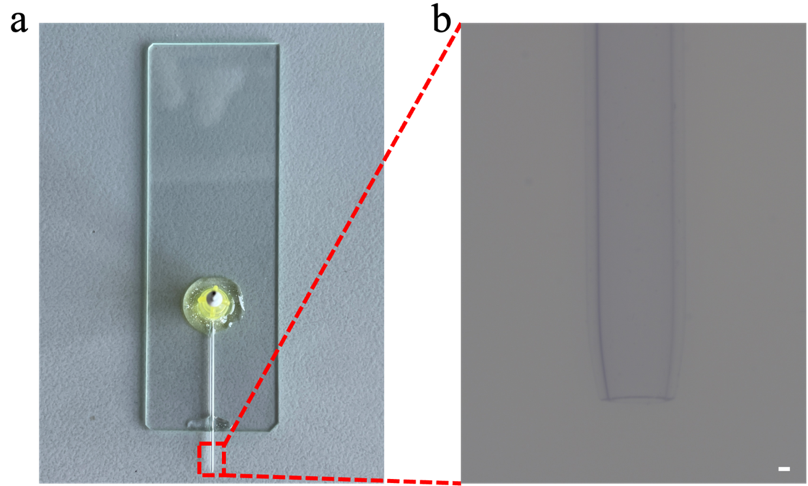
**

***Figure S2.*** *The capillary microfluidic device used to extrude Gel-Okra@PNS scaffolds: (a) photograph showing the microfluidic device; (b) microscopic image of the tip of the capillary. The scale bar is 100 µm.*

*
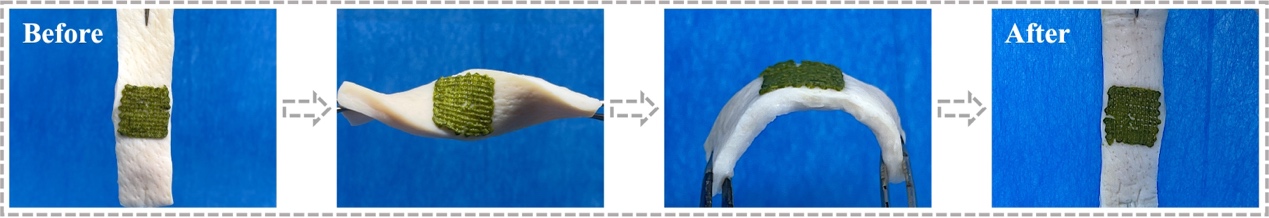
*

***Figure S3.*** *Photographs showing that the Gel-Okra@PNS adhered firmly to the pigskin after being twisted and bent.*
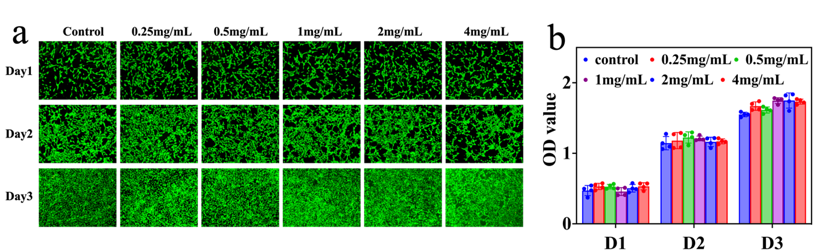


***Figure S4.*** *(a) (b) 3T3 cells were subjected to live/dead staining and CCK-8 assay after incubation with Gel-Okra@PNS extract for different days. The concentrations in the figure represent the leaching solutions obtained by immersing scaffolds weighing 0.25mg, 0.5mg, 1mg, 2mg, and 4mg in 1 ml of culture medium.*

***
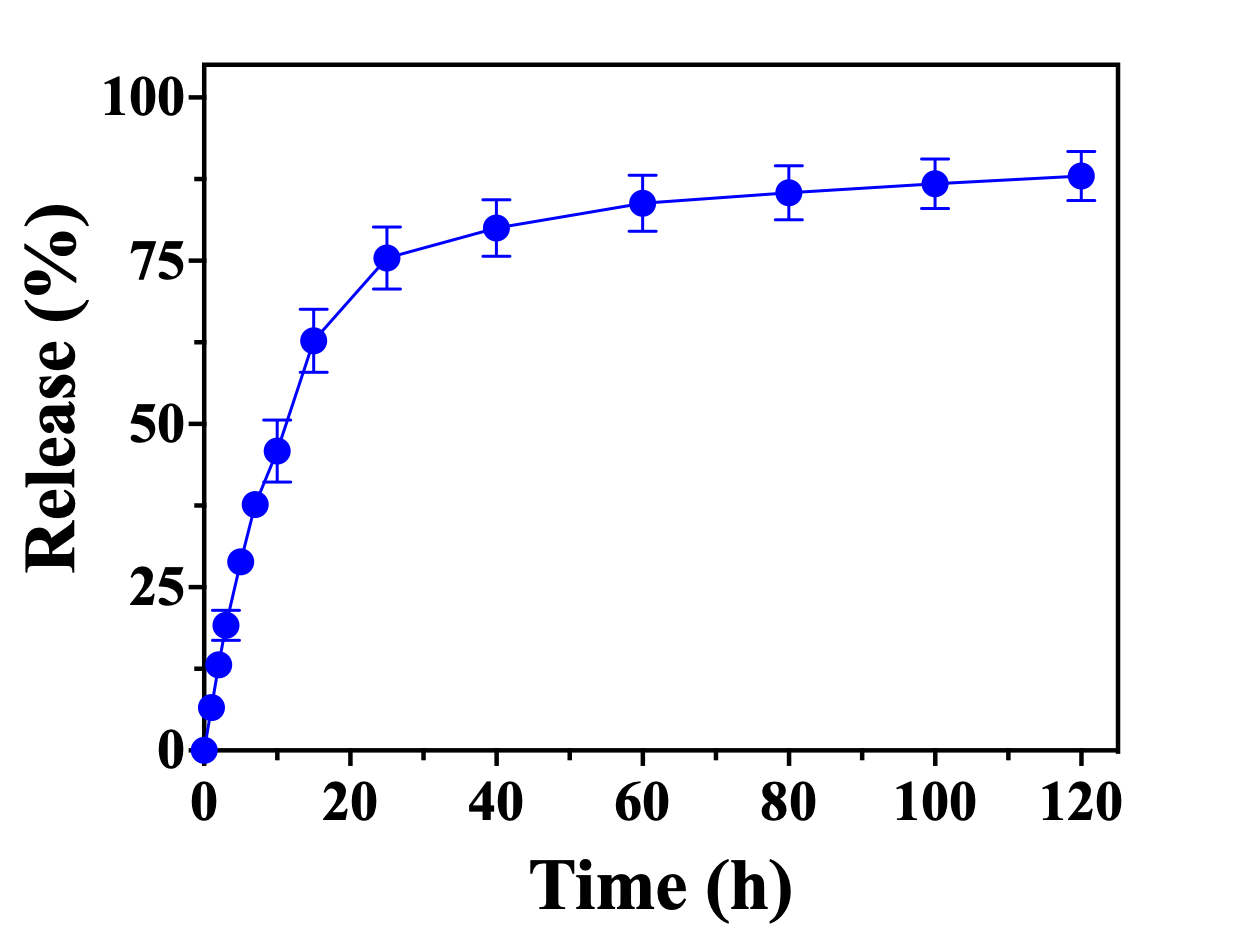
***

***Figure S5.*** *The in vitro release profile of PNS from the Gel-Okra@PNS scaffold.*

*
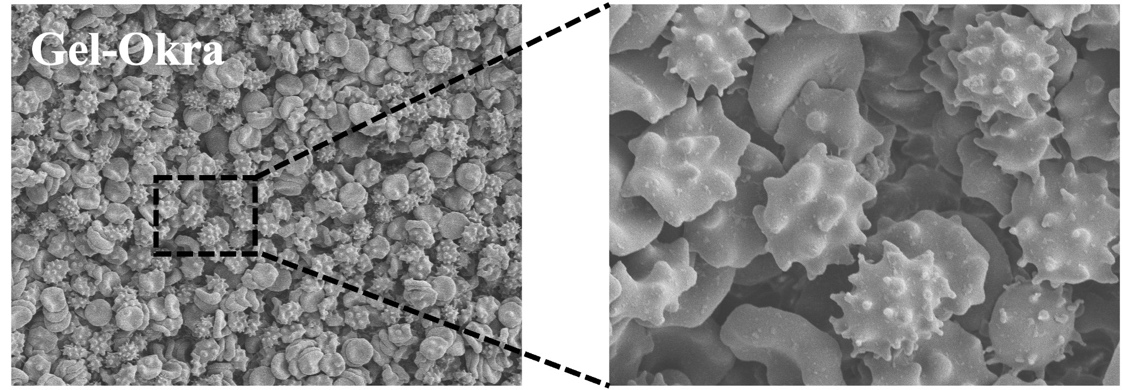
*

***Figure S6.*** *The SEM image illustrating the Gel-Okra scaffold mixed with whole blood.*

**
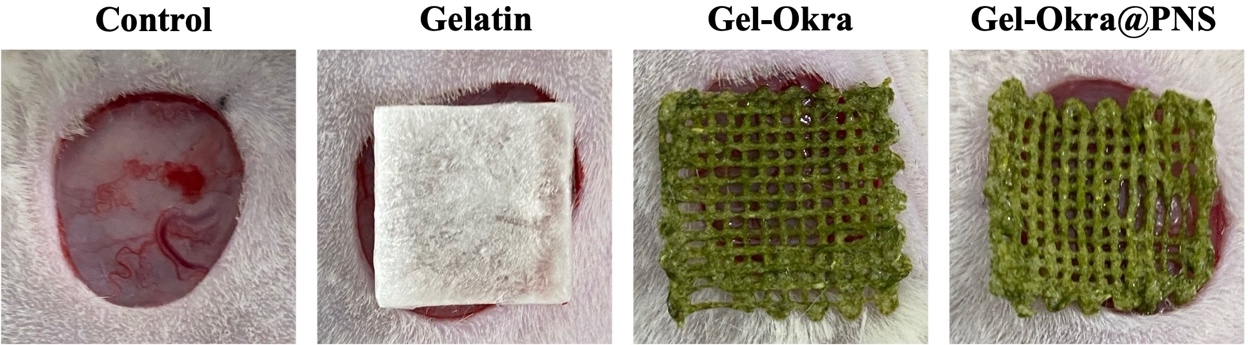
**

***Figure S7.*** *Photographs showing various treatments in each group of the in vivo wound healing experiments.*

***Table 1.*** *Various coagulation indicators of blood supernatant.*

| Group | PT (s) | APTT (s) | FIB LIQ (g/l) | TT (s) |
| --- | --- | --- | --- | --- |
| Control | 11.8 | 33.9 | 2.91 | 17.3 |
| Gelatin | 11.6 | 30.3 | 2.94 | 16.2 |
| Gel-Okra | >120 | >180 | <0.6 | >240 |
| Gel-Okra@PNS | >120 | >180 | <0.6 | >240 |

*
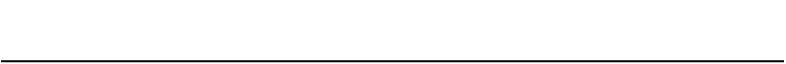
*
